# 30-Year Trends in the Incidence, Characteristics, and Outcome of Cardiac Sarcoidosis in a Nationwide Cohort

**DOI:** 10.1016/j.jacadv.2024.101102

**Published:** 2024-07-13

**Authors:** Pauli Pöyhönen, Jukka Lehtonen, Diana Velikanova, Piia Simonen, Valtteri Uusitalo, Henriikka Mälkönen, Hanna-Kaisa Nordenswan, Tapani Vihinen, Kari Kaikkonen, Petri Haataja, Tuomas Kerola, Tuomas T. Rissanen, Ville Vepsäläinen, Aleksi Alatalo, Päivi Pietilä-Effati, Markku Kupari

**Affiliations:** a Heart and Lung Center, Helsinki University Hospital and University of Helsinki, Helsinki, Finland; b Radiology, Helsinki University Hospital and University of Helsinki, Helsinki, Finland; c Clinical Physiology and Nuclear Medicine, Helsinki University Hospital and University of Helsinki, Helsinki, Finland; d Heart Center, Turku University Hospital, Turku, Finland; e Medical Research Center Oulu, University and University Hospital of Oulu, Oulu, Finland; f Heart Hospital, Tampere University Hospital, Tampere, Finland; g Department of Internal Medicine, Päijät-Häme Central Hospital, Lahti, Finland; h Heart Center, North Karelia Central Hospital, Joensuu, Finland; i Heart Centre, Kuopio University Hospital, Kuopio, Finland; j South Ostrobothnia Central Hospital, Seinäjoki, Finland; k Vaasa Central Hospital, Vaasa, Finland

**Keywords:** cardiac sarcoidosis, diagnostic imaging, incidence, phenotype, prognosis

## Abstract

**Background:**

Cardiac sarcoidosis (CS) is a rare but potentially fatal inflammatory cardiomyopathy.

**Objectives:**

The authors studied temporal changes in the incidence, characteristics, and outcome of CS.

**Methods:**

A retrospective analysis was made of a 30-year nationwide cohort of CS.

**Results:**

The cohort comprised 511 patients with a median age of 52 years and female preponderance (69%). Altogether 77, 166, and 268 cases of CS were diagnosed in years 1988 to 2009, 2010 to 2014, and 2015 to 2019, respectively; the 5-year count of 2015 to 2019 was 134-fold the count of 1990 to 1994 (268/2) and 18-fold the count of 2000 to 2004 (268/15). Prior to 2010, compared with the later periods, CS presented more often with ventricular tachycardia/fibrillation (prevalence 36% vs 19% in 2010-2014 and 11% in 2015-2019, *P* < 0.001), left ventricular ejection fraction <50% (49%, 35%, and 31%; *P* = 0.010), and elevation of natriuretic peptides (87%, 57%, and 49%; *P* < 0.001). On magnetic resonance imaging, late gadolinium enhancement involved a median of 15% (IQR: 11%-22%) of left ventricular mass in studies of 1988 to 2009 (n = 16), 15% (IQR: 9%-22%) in studies of 2010 to 2014 (n = 87), and 11% (IQR: 5%-19%) in studies of 2015 to 2019 (n = 150) (*P* = 0.031). The respective 5-year incidences of the composite of death, heart transplantation, left ventricular–assisted device implantation, or ventricular tachyarrhythmia were 40% (95% CI: 29%-51%), 32% (95% CI: 25%-39%), and 23% (95% CI: 16%-30%) (*P* = 0.002). The prognostic trend disappeared after adjustment for differences in the presenting phenotype.

**Conclusions:**

Diagnoses of incident CS have increased exponentially in Finland. Concurrently, the phenotype has turned milder and prognosis better, suggesting detection of CS at an earlier stage of its course.

Sarcoidosis is an etiologically unknown granulomatous inflammation that can involve the heart as part of a multiorgan disease or, more rarely, as an isolated cardiomyopathy.^1^ Granulomas can invade any cardiac structure causing silent or symptomatic injury, scarring, and dysfunction.^1^^,^^2^ When clinically manifest, cardiac sarcoidosis (CS) typically appears as high-grade heart block, ventricular tachyarrhythmia, or new-onset heart failure (HF).^2^^,^^3^ Its most ominous manifestation is sudden cardiac death (SCD) with a risk that approximates 10% over 5 years in clinically manifest CS^4^ and involves subclinical cases as well.^5^

CS is considered an orphan condition, but knowledge of its epidemiology remains imprecise due to its multiple and often subclinical manifestations, racial and geographic differences, and the diversity of its diagnostic criteria across years, countries, and institutions.^6^, ^7^, ^8^, ^9^ Experts are divided on the diagnostic role of sarcoidosis histology, which some consider essential for diagnosis^6^^,^^7^ while others downgrade, diagnosing CS even from clinical and imaging findings alone.^8^^,^^10^, ^11^, ^12^ For all diagnostic issues, recent observations suggest increases in admissions and need of treatment for clinically manifest CS.^3^^,^^13^, ^14^, ^15^ It is presumed that heightened clinical awareness of CS and wider use of advanced cardiac imaging have facilitated the detection of CS.^3^^,^^14^^,^^15^ Whether trends like that have translated into changes in the characteristics of CS at the time of diagnosis or improvement in prognosis has not been studied hitherto. The ongoing nationwide registry of MIDFIN (Myocardial Inflammatory Diseases in Finland)^1^^,^^3^^,^^5^ includes patients with clinically manifest and histologically proven CS diagnosed since the late 1980s. For the present work, we analyzed temporal trends in the presenting manifestations, diagnostics, treatment, and outcome of patients entered into the registry until the end of 2019.

## Methods

### Study population

At the end of 2019, the MIDFIN registry included 594 cases of biopsy-proven CS detected in our country since 1988. Of them, we included in the present analysis 511 patients with clinical diagnosis, treatment, and surveillance in the hospitals of the MIDFIN network. Cases diagnosed postmortem (n = 64) or post-transplantation (n = 14) or having poor data quality (n = 5) were excluded from the main analyses. The post-transplant diagnoses were counted, however, in analyzing CS-related heart transplantations. All patients met the diagnostic criteria of the Heart Rhythm Society,^6^ including proof of sarcoidosis histology. Throughout the period covered by our study, endomyocardial biopsy (EMB) was the preferred diagnostic procedure to verify the presence of granulomatous inflammation.^16^

The MIDFIN registry study was approved by a national ethical review board in 2009 (STM/1219/2009). Written informed consent was obtained from each patient alive at the time of recruitment into the registry.

### Data acquisition

Most of the data analyzed here were extracted from the MIDFIN registry, the details of which have been described in our prior publications.^1^^,^^3^^,^^5^ The cases of CS were identified in retrospect from the late 1980s till 2010 but have since been included mainly prospectively without systematic cardiac screening of asymptomatic patients with sarcoidosis. The case particulars have been derived from hospital charts and other medical documents and entered into the internet-based registry by cardiologists and research nurses in the participating hospitals. The MIDFIN database includes granular information on patients’ demographics, clinical cardiac manifestations, associated diseases, diagnostic imaging studies, biopsies, and routine laboratory studies including assessment of cardiac biomarkers. The dates of presentation and diagnosis of CS were defined by the first medical contact due to cardiac symptoms and the fulfillment of the Heart Rhythm Society criteria,^6^ respectively. Their difference in months was taken as the diagnostic delay in our analyses. Further entries involve details of treatment with drugs, devices, catheter-based procedures, and surgery, as well as the occurrence and specifics of adverse cardiac and noncardiac events as verified and recorded by cardiologists responsible for the care of CS in their respective hospitals. The data concerning the number of heart transplantations in Finland and the results of histopathologic studies of the explanted hearts were retrieved from the national heart transplantation registry at Helsinki University Hospital.

### Review of cardiac imaging studies

The data on echocardiographic left ventricular ejection fraction used in the present analyses were taken from the reports prepared by the attending cardiologist as part of routine diagnostics at or after the patient’s hospital admission. In contrast, cardiac magnetic resonance imaging (CMRI) studies, ^18^F-fluorodeoxyglucose positron emission tomography (^18^F-FDG-PET) scans, and single-photon emission computed tomography perfusion studies done prior to diagnosis were acquired for reanalysis at the MIDFIN core center (Helsinki University Hospital). Studies failing an introductory quality check were rejected from further analysis. The details of the imaging techniques and the methods of reanalyses are described in the Supplemental Material.

### Definition of outcome endpoints

The primary variable for outcome assessment was the time from diagnosis to the composite endpoint of death, heart transplantation, implantation of a left ventricular assist device (LVAD), or life-threatening ventricular arrhythmia defined as either ventricular fibrillation (VF) defibrillated externally or by an implantable cardioverter-defibrillator (ICD) or ventricular tachycardia (VT) converted by an ICD, external synchronized cardioversion, or amiodarone infusion. Other endpoints were the composite of death, transplantation, or LVAD implantation; and the composite of SCD, nonfatal VF, or VT as defined above. The causes of death were ascertained from clinical and autopsy reports. Mortality data were double-checked from the files of the Digital and Population Data Services Agency of Finland. The patients were followed up for all events till the end of 2020.

### Statistical analyses

Continuous variables are presented as mean ± SD and median (IQR) for normally distributed and skewed data, respectively. Categorical variables are reported as frequencies and percentages. Group comparisons of continuous data were made with 1-way analysis of variance or Kruskal-Wallis test, as appropriate, while frequencies were compared using the chi-square test or Fisher exact test. Follow-up times were calculated from the date of diagnosis to the first endpoint event or conclusion of follow-up. Cumulative incidence analysis^17^ was used to calculate unadjusted incidence estimates and to construct incidence-time curves and their 95% CIs; the Gray test^18^ was used for comparisons between groups. Cox regression analysis was used to assess the predictors of the primary outcome based on HRs and 95% CIs. In analyzing the composite incidence of SCD, VF, or VT (secondary arrhythmic endpoint, see above), LVAD implantation, heart transplantation and death from HF or noncardiac cause were considered competing events. Cox regression analysis was used to assess the predictors of outcome based on HRs and 95% CIs. The proportionality of hazards was ascertained by analyzing Schoenfeld residuals. Only cases with complete data were included in the incidence analyses. *P* values <0.05 were considered statistically significant. Analyses were performed using the R (version 4.0.4, R Foundation).

## Results

### The rate and quality characteristics of CS diagnoses

**Figure 1:**
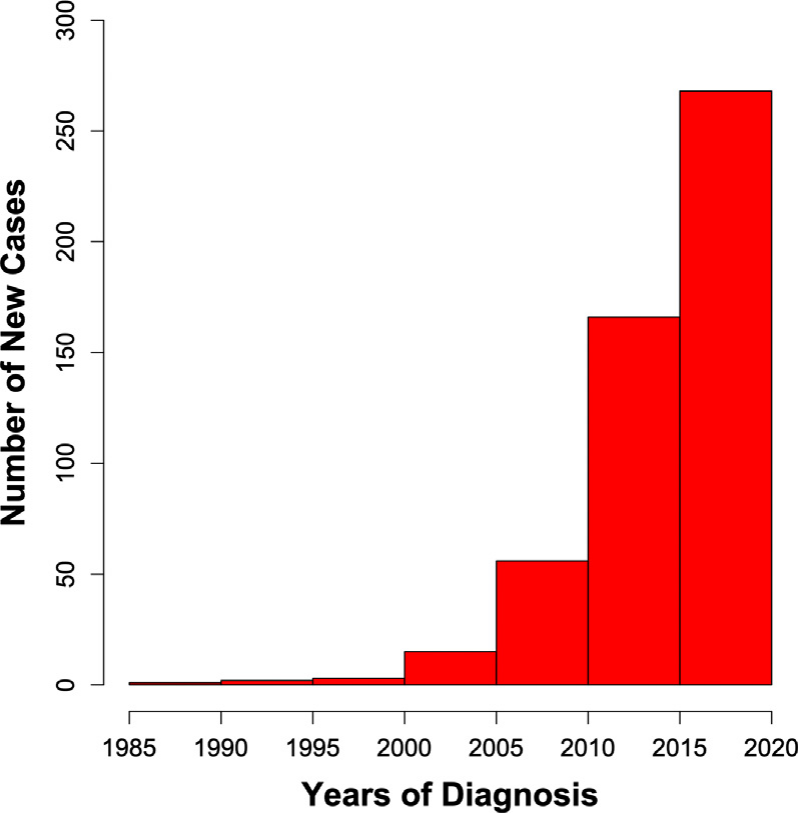
**Five-Year Rates of Incident Cases of Clinically Manifest Cardiac Sarcoidosis Diagnosed in Finland since the Late 1980s** The upsurge of new diagnoses started after the turn of the millennium and has continued ever since.

Figure 1 shows the 5-year counts of incident cases of CS diagnosed in Finland from the late 1980s till the end of 2019 by the MIDFIN registry. The upsurge of cases started after the turn of the millennium and has continued ever since. The 5-year count of 2015 to 2019 (n = 268) translates into a crude CS incidence of 1.2/y/100,000 adults (>18 years) and is 134-fold (268/2) the count of 1990 to 1994, 18-fold (268/15) the count of 2000 to 2004, and 1.6-fold (268/166) the count of 2010 to 2014.

The diagnosis of CS was definite, that is, confirmed by myocardial histology,^6^ in 225 of the 511 patients (44%), the rest having probable CS^6^ with sarcoid granulomas proven from samples of lymph nodes (n = 200) or extracardiac organs (n = 86). Analyzed by the years of diagnosis, 53/77 patients (69%) diagnosed in 1988 to 2009 had definite CS compared with 74/166 (45%) and 98/268 (37%) diagnosed in 2010 to 2014 and 2015 to 2019, respectively (*P* < 0.001). The rate of diagnostic EMBs decreased across the above periods from 69/75 (92%) to 124/156 (79%) and further to 173/234 (74%) (*P* = 0.004). The median delay from presentation to diagnosis of CS was 3.5 months (IQR: 0.7-16.9) in cases diagnosed prior to 2010 vs 4.4 months (IQR: 1.5-16.5) and 3.0 months (IQR: 1.3-7.3) in cases in 2010 to 2014 and 2015 to 2019, respectively (*P* = 0.040). Analyzed by the main presenting cardiac manifestation (see Table 1), the diagnostic delay was 1.5 months (IQR: 0.4-6.8) for ventricular tachyarrhythmias, 2.9 months (IQR: 0.8-7.6) for HF, 4.0 months (IQR: 1.7-16.5) for high-grade atrioventricular block, and 4.0 months (IQR: 2.0-7.1) for the other manifestations combined (*P* < 0.001).

**Table 1:** Patient Characteristics at Presentation of Manifest Cardiac Sarcoidosis

	All Patients (N = 511)	Patients by the Year of Diagnosis			
		1988-2009 (n = 77)	2010-2014 (n = 166)	2015-2019 (n = 268)	*P* Value
Age, y	52 (43-58)	51 (44-56)	51 (41-57)	53 (45-59)	0.088
Female	355 (69)	55 (71)	123 (74)	177 (66)	0.192
Body mass index, kg/m^2^	26 (24-30), n = 493	26 (23-30), n = 75	26 (24-29), n = 158	27 (24-30), n = 260	0.062
Main presenting manifestation					<0.001
High-grade atrioventricular block	266 (52)	30 (39)	86 (52)	150 (56)	
Heart failure	82 (16)	12 (16)	25 (15)	45 (17)	
Ventricular tachyarrhythmia^a^	90 (18)	28 (36)	32 (19)	30 (11)	
Other^b^	73 (14)	7 (9)	23 (14)	43 (16)	
Selected laboratory findings					
LVEF on echocardiography, %	55 (45-60)	50 (40-55)	55 (43-60)	55 (45-62)	<0.001
<50%	178 (35)	38 (49)	58 (35)	82 (31)	0.010
Elevated cardiac troponins^c^	243 (52), n = 471	32 (49), n = 65	79 (53), n = 148	132 (51), n = 258	0.838
Elevated natriuretic peptides^d^	228 (55), n = 416	33 (87), n = 38	79 (57), n = 139	116 (49), n = 239	<0.001
eGFR, mL/min/1.73 m^2^	84 (70-97), n = 488	86 (77-95), n = 74	89 (73-100), n = 152	81 (67-96), n = 262	0.025
Associated diseases					
History of extracardiac sarcoidosis^e^	87 (17)	15 (19)	26 (16)	46 (17)	0.759
Diabetes	40 (8)	4 (5)	18 (11)	18 (7)	0.193
Hypertension	139 (27)	19 (25)	41 (25)	79 (29)	0.479
Coronary heart disease^f^	40 (8)	8 (10)	15 (9)	17 (6)	0.396

Values are median (IQR) or n (%). *P* values pertain to comparisons across the 3 groups by Kruskal-Wallis test, chi-square test or Fisher exact test, as appropriate.

eGFR = estimated glomerular filtration rate (by the CKD-EPI formula); LVEF = left ventricular ejection fraction.

a Sustained ventricular tachycardia or ventricular fibrillation terminated by external defibrillation.

b Other manifestations include frequent ventricular premature beats or nonsustained ventricular tachycardia; mimics of acute myocardial ischemia; anginal chest pain; syncope; and miscellaneous symptoms and findings including breathlessness, fatigue, pericardial effusion, and abnormalities on 12-lead electrocardiogram.

c Troponin T by the Elcsys immunoassay (Roche Diagnostics, Germany) exceeding the contemporaneous reference range (ie, being ≥0.03 μg/L by the 4th generation assay or ≥15 ng/L by the 5th generation assay); or hs-troponin I ≥50 ng/l.

d Circulating brain natriuretic peptide >100 ng/L or N-terminal brain natriuretic propeptide >400 ng/L.

e Based on prior medical documents and clinical assessment at presentation.

f History of clinical coronary heart disease at presentation, or coronary artery stenosis exceeding 50% by selective angiography (done in 251 of 511 patients).

### Patient characteristics

Table 1 summarizes the key presenting characteristics for the entire cohort and the 3 subgroups formed by the years of confirmed diagnosis. The data show statistically significant differences across the subgroups in the presenting cardiac manifestations and in the echocardiographic and laboratory biomarkers of myocardial involvement. Accordingly, compared with patients diagnosed in 2010 to 2014 or 2015 to 2019, the group diagnosed prior to 2010 had more ventricular tachyarrhythmias and less high-grade heart block and miscellaneous other cardiac symptoms at presentation (*P* < 0.001), together with poorer left ventricular ejection fraction (*P* < 0.001) and more frequent elevation of circulating natriuretic peptides (*P* < 0.001). There were no statistically significant differences in the demographic characteristics or in the prevalence of associated diseases including history of prior extracardiac sarcoidosis (Table 1).

Table 2 details the numbers of diagnostic examinations in the domain of advanced cardiac imaging and summarizes the results of studies that could be reanalyzed (see Supplemental Methods). The median time intervals from presentation were 19 days (IQR: 3-77) for CMRI, 64 days (IQR: 22-209) for PET scans, and 71 days (IQR: 26-254) for single-photon emission computed tomography scans. The data show, first, that much higher proportions of patients diagnosed after 2009 than earlier had undergone ^18^F-FDG-PET and CMRI, the frequency of single-photon emission computed tomography perfusion scans being independent of the period of diagnosis and low overall. Second, all imaging modalities expose a change with time toward decreasing severity of myocardial involvement. Of note, both left and right ventricular involvement on CMRI (ejection fraction, extent of late gadolinium enhancement) differ even between the groups of 2010 to 2014 and 2015 to 2019, suggesting less extensive myocardial involvement in incident CS diagnosed in the latter half of the past decade.

**Table 2:** Findings on Advanced Imaging Prior to the Diagnosis of Cardiac Sarcoidosis

	All Patients (N = 511)	Patients by the Year of Diagnosis			
		1988-2009 (n = 77)	2010-2014 (n = 166)	2015-2019 (n = 268)	*P* Value
Whole-body^18^F-FDG PET	167 (33)	0	52 (31)	112 (42)	<0.001
Cardiac^18^F-FDG PET					
Done	231 (45)	15 (19)	78 (47)	138 (51)	<0.001
Analyzable	191 (37)	6 (8)	59 (36)	126 (47)^a^	<0.001
Abnormal myocardial FDG uptake	181 (95)	6 (100)	57 (97)	118 (94)	0.644
LV segments with FDG uptake	6 (3-9), n = 190	8 (6-9)	7 (4-10)	5 (3-8), n = 125^a^	0.034
CMRI					
Done	327 (64)	30 (39)	113 (68)	184 (69)	<0.001
Analyzable	285 (56)	24 (31)	101 (61)	160 (60)	<0.001
Left ventricular LGE	265 (96), n = 277	21 (100), n = 21	92 (96), n = 96	152 (95), n = 160	0.903
Right ventricular free wall LGE	105 (39), n = 266	12 (71), n = 17	40 (44), n = 90	53 (33), n = 159	0.006
Left ventricular LGE mass, %^b^	14 (7-21), n = 253	15 (11-22), n = 16	15 (9-22), n = 87	11 (5-19), n = 150^a^	0.031
Left ventricular ejection fraction, %	48 (39-56), n = 281	44 (40-47), n = 21	45 (32-54), n = 100	51 (41-59), n = 160^a^	0.001
Right ventricular ejection fraction, %	55 (46-61), n = 270	53 (47-56), n = 15	52 (43-58), n = 96	57 (50-63), n = 159^a^	<0.001
SPECT					
Done	141 (28)	16 (21)	52 (31)	73 (27)	0.227
Analyzable	136 (27)	16 (21)	50 (30)	70 (26)	0.298
Patients with perfusion defect	120 (88)	15 (94)	45 (90)	60 (86)	0.692
Summed rest score of segments with perfusion defect	5 (3-10)	9 (5-14)	7 (4-12)	5 (2-10)	0.085

Values are n (%) or median (IQR). *P* values pertain to comparisons across the 3 groups by Kruskal-Wallis, chi-square test, or Fisher exact test, as appropriate.

CMRI = cardiac magnetic resonance imaging; FDG = fluorodeoxyglucose; LGE = late gadolinium enhancement; PET = positron emission tomography; SPECT = single-photon emission computed tomography.

a *P* <0.05 for comparison with patients diagnosed in 2010 to 2014.

b LGE mass by the full width at half maximum method as percentage of LV mass.

### Treatment and outcome

Table 3 summarizes the use of drugs and devices in our study cohort. Nearly all patients (98%) received corticosteroids for immunosuppression. Patients diagnosed prior to 2010 received more often adjunctive immunosuppressants (azathioprine, cyclosporin) and drugs for HF, but no other differences were noted across the subgroups. The rate of ICD implantations was 76% and did not vary by the years of diagnosis.

**Table 3:** Summary of Treatment From Diagnosis to End of Follow-Up

	All Patients (N = 511)	Patients by the Year of Diagnosis			
		1988-2009 (n = 77)	2010-2014 (n = 166)	2015-2019 (n = 268)	*P* Value
Immunosuppression					
Corticosteroids	500 (98)	76 (99)	163 (98)	261 (97)	0.842
Pulse steroid therapy	20 (4)	3 (4)	6 (4)	11 (4)	1.000
Azathioprine	185 (36)	44 (57)	69 (42)	72 (27)	<0.001
Methotrexate	57 (11)	9 (12)	22 (13)	26 (10)	0.514
Mycophenolate mofetil	33 (6)	8 (10)	11 (7)	14 (5)	0.245
Cyclosporin	30 (6)	13 (17)	13 (8)	4 (1)	<0.001
Infliximab	27 (5)	1 (1)	8 (5)	18 (7)	0.152
Drugs and interventions for heart failure and arrhythmias					
Beta-adrenergic blockers	483 (95)	75 (97)	158 (95)	250 (93)	0.378
ACEI, ARB, or sacubitril/valsartan	353 (69)	59 (77)	118 (71)	176 (66)	0.148
Mineralocorticoid receptor antagonist	160 (31)	30 (39)	61 (37)	69 (26)	0.016
Loop diuretic	160 (31)	33 (43)	59 (36)	68 (25)	0.005
Digoxin	13 (3)	6 (8)	3 (2)	4 (1)	0.006
Cardiac resynchronization therapy	129 (25)	19 (25)	50 (30)	60 (22)	0.196
Implantable cardioverter-defibrillator	390 (76)	59 (77)	127 (77)	204 (76)	0.994
Dual chamber pacemaker	332 (65)	47 (61)	108 (65)	177 (66)	0.719

Values are n (%) receiving the specified therapy any time from presentation to end of follow-up. *P* values refer to comparisons between the 3 groups by Kruskal-Wallis or chi-square test.

ACEI = angiotensin converting enzyme inhibitor; ARB = angiotensin receptor blocker.

**Figure 2:**
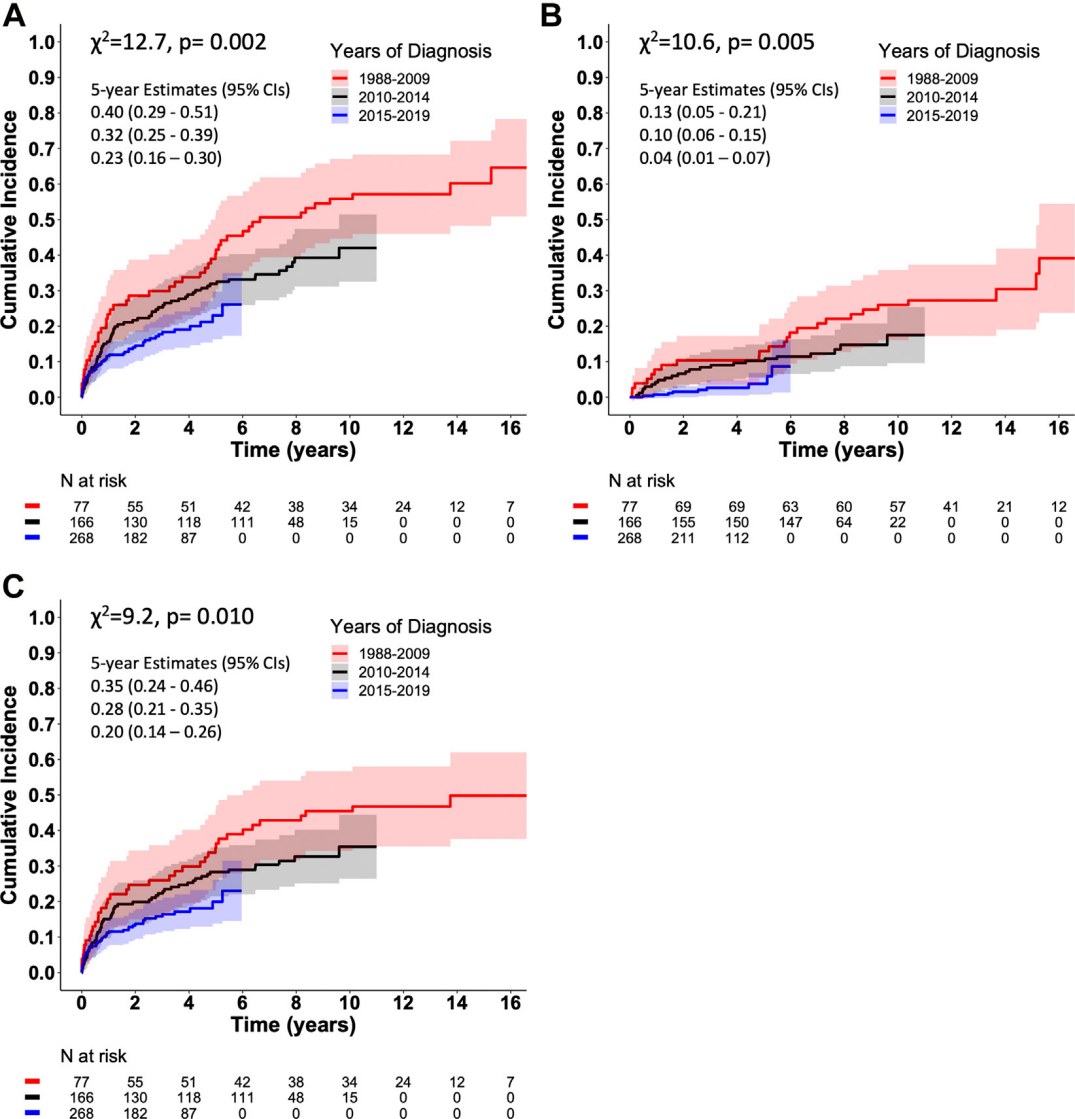
**Evolution in the Prognosis of Cardiac Sarcoidosis Over the Last 3 Decades in Finland** Cumulative incidence graphs by the periods of diagnosis years for (A) the composite of death, heart transplantation, left ventricular assist device (LVAD) implantation, ventricular fibrillation (VF), or ventricular tachycardia needing therapy (VT); (B) the composite of death, transplantation, or LVAD implantation; and (C) the composite of sudden cardiac death, VF, or VT with HF-death, noncardiac death, transplantation and LVAD implantation considered competing events. The improvement of prognosis over time is statistically significant in arrhythmic and non-arrhythmic events alike.

Table 4 lists all cardiac events recorded in the 511-patient cohort until the end of 2020. The primary outcome endpoint (n = 159) was composed of 24 deaths, 7 transplantations, 2 LVAD implantations, and 126 episodes of VF or VT as first events during a median follow-up of 4.0 years (IQR: 1.9-6.5). The estimated 5-year and 10-year cumulative incidences in the entire cohort were 29% (95% CI: 25%-34%) and 42% (95% CI: 36%-48%), respectively. Figure 2A shows the incidence graphs for the primary endpoint in patients stratified by the years of CS diagnosis. The 5-year incidence estimates were 40% (95% CI: 29%-51%), 32% (95% CI: 25%-39%), and 23% (16%-30%) in patients diagnosed in 1988 to 2009, 2010 to 2014, and 2015 to 2019, respectively (*P* = 0.002). Comparable graphs for the composite of death, transplantation, or LVAD implantation are shown in Figure 2B, while Figure 2C shows the graphs for the occurrence of SCD, VF, or VT with HF-death, noncardiac death, transplantation, and LVAD analyzed as competing events. The incidence of SCD (fatal and aborted combined) per 100 patient-years was 2.1 (IQR: 1.6-2.7) overall, being 2.4 (IQR: 1.4-3.7) in patients diagnosed prior to 2010 and 1.7 (IQR: 0.9-2.7) in those diagnosed in 2015 to 2019.

**Table 4:** Outcome Events in 511 Patients With Cardiac Sarcoidosis

Death	43
From terminal heart failure	9
Sudden cardiac	11
Following heart transplantation or LVAD implantation	8
Noncardiac	15
Heart transplantation	21
LVAD implantation	3
Bridge to transplantation	1
Destination therapy	2
Ventricular tachyarrhythmia	126
Ventricular fibrillation (aborted sudden cardiac death)	48
Ventricular tachycardia only^a^	78

Values are n.

LVAD = left ventricular assist device.

a Converted by intracardiac cardioverter-defibrillator, external synchronized cardioversion, or amiodarone infusion; self-terminated episodes were not counted.

Table 5 shows the results of Cox regression analyses on the candidate predictors of the primary outcome endpoint. The year of CS diagnosis predicted the outcome statistically significantly in univariate analysis both as a continuous and a categorical variable. It lost, however, its prognostic significance in multivariable analyses, where presentation with ventricular tachyarrhythmia was the most consistent predictor, with left ventricular ejection fraction, elevation of cardiac troponins, and diagnosis by myocardial histology having variable independent effects depending on the model.

**Table 5:** Results of Cox Regression Analyses for the Prediction of the Occurrence of Outcome Events^a^ in 511 Patients With Cardiac Sarcoidosis

	Univariable Analyses			Multivariable Models			
				Model 1 (e = 159, n = 511)		Model 2 (e = 145, n = 471)	
	Events/N	HR (95% CI)	*P* Value	HR (95% CI)	*P* Value	HR (95% CI)	*P* Value
Age, per 1 y	159/511	0.99 (0.97-1.00)	0.083	0.99 (0.98-1.01)	0.240	0.99 (0.97-1.01)	0.200
Female	159/511	0.92 (0.66-1.29)	0.630				
Year of diagnosis since 1988, per +10 y	159/511	0.58 (0.43-0.77)	<0.001				
Diagnostic period	159/511		0.002		0.480		0.290
1988-2009 (reference)		NA	NA	NA	NA	NA	NA
2010-2014		0.66 (0.44-0.97)	0.033	0.91 (0.60-1.36)	0.630	0.85 (0.56-1.30)	0.450
2015-2019		0.47 (0.31-0.71)	<0.001	0.76 (0.49-1.19)	0.230	0.69 (0.44-1.10)	0.120
Diagnostic delay, per 1 mo from presentation	159/511	1.00 (0.99-1.01)	0.910				
Definite diagnosis from myocardial biopsy	159/511	2.17 (1.57-2.99)	<0.001	1.66 (1.18-2.34)	0.004	1.40 (0.98-2.00)	0.063
Main presenting manifestation	159/511		<0.001		<0.001		<0.001
atrioventricular block (reference)		NA	NA	NA	NA	NA	NA
heart failure		1.91 (1.23-2.98)	0.004	1.19 (0.68--2.08)	0.540	1.16 (0.65-2.07)	0.610
ventricular tachyarrhythmia		3.54 (2.43-5.17)	<0.001	2.60 (1.72-3.94)	<0.001	2.43 (1.57-3.77)	<0.001
other^b^		1.10 (0.64-1.91)	0.720	1.13 (0.66-1.96)	0.650	1.03 (0.57-1.87)	0.920
LVEF on echocardiography, per +10%	159/511	0.75 (0.67-0.84)	<0.001	0.81 (0.69-0.95)	0.011	0.81 (0.69-0.96)	0.016
Elevation of cardiac troponins^b^	145/471	2.23 (1.58-3.15)	<0.001			1.79 (1.23-2.61)	0.002
Elevation of natriuretic peptides^b^	125/416	2.03 (1.37-3.00)	<0.001				

LVEF = left ventricular ejection fraction; NA = not applicable.

a Composite of death, heart transplantation, left ventricular assist device implantation, or ventricular tachyarrhythmia (see Methods for details).

b For details, see the footnotes in Table 1.

### Heart transplantations in CS

**Figure 3:**
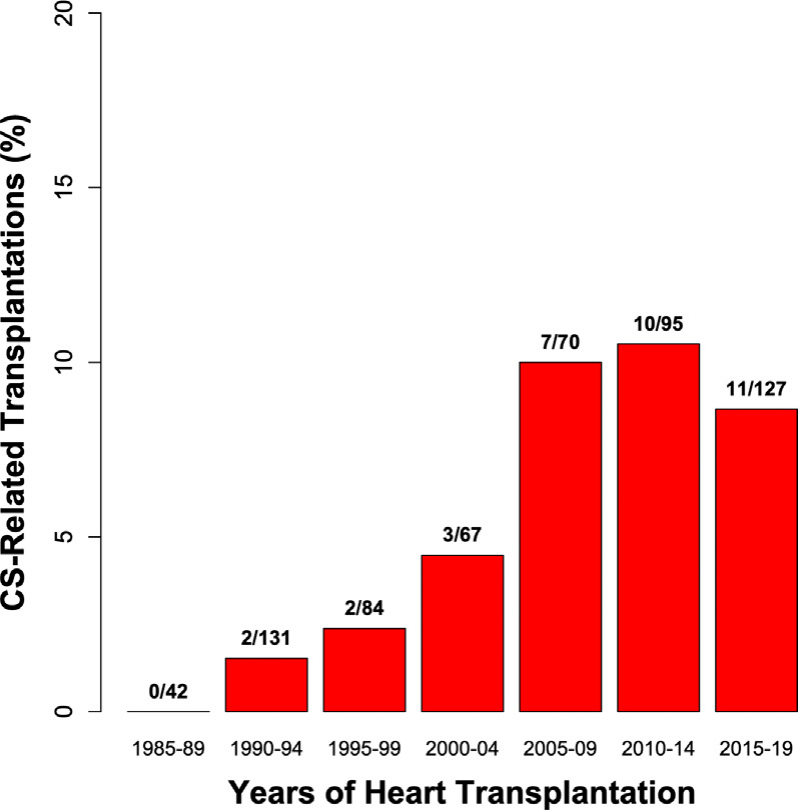
**The Changing Proportion of Heart Transplantations for CS** The columns show the 5-year proportions of CS-related heart transplantations since the late 1980s in Finland. The exact counts of CS-related and all transplantations are given above the columns. Of the 35 cases of CS, 20 had been diagnosed clinically and were confirmed post-transplantation and 15 were detected only at the histopathologic study of the explanted heart. Both the number of CS-related transplantations and their share of cardiac transplant surgery grew markedly over the 30-year coverage of our analysis.

Between 1985 and end of 2019, altogether 616 patients underwent heart transplantation in Finland. Of them, 35 (5.7%) had CS, which was diagnosed clinically and confirmed post-transplantation in 20 cases and found only at the histopathologic study of the explanted heart in the rest 15 patients. Figure 3 shows that both the number of CS-related transplantations and their share of cardiac transplant surgery grew markedly over the 30-year coverage of our work. Since 2005, close to 10% of all heart transplantations in Finland have been done for terminal HF and/or therapy-resistant ventricular tachyarrhythmias caused by clinically diagnosed or missed CS.

## Discussion

Our study shows that the increase in the number of incident cases of clinically manifest CS in Finland, that we came across in the early 2010s,^3^ has continued, the successive 5-year rates of new diagnoses demonstrating an exponential growth since the late 1980s (Figure 1). The other key findings, summarized in the Central Illustration, are the increase in the diagnostic use of CMRI and ^18^F-FDG-PET, and the CS phenotype at diagnosis turning milder with a reduced frequency of life-threatening arrhythmias and less myocardial inflammation, scarring, and dysfunction. Finally, the outcome of CS improved without major evolution in the CS-targeted therapy, but the prognostic trend disappeared after adjustment for the presenting phenotype and severity of cardiac involvement.

**Central Illustration:**
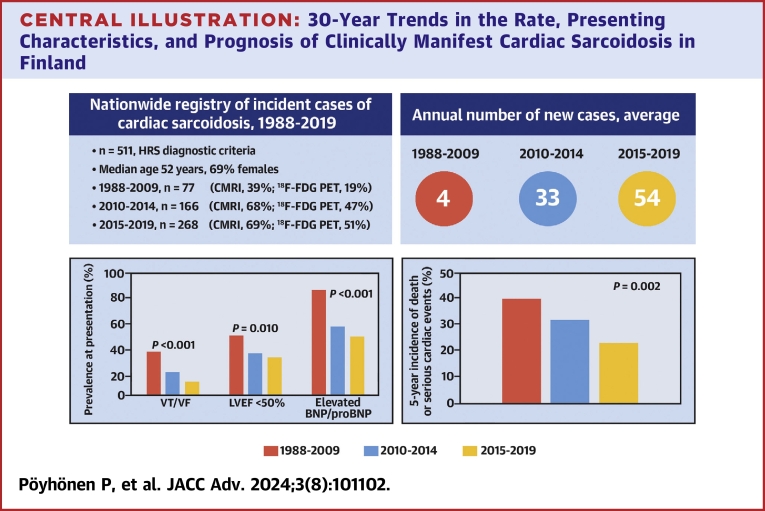
**30-Year Trends in the Rate, Presenting Characteristics, and Prognosis of Clinically Manifest Cardiac Sarcoidosis in Finland** The average annual number of incident cases grew markedly over time as did the proportion of patients undergoing cardiac magnetic resonance imaging (CMRI) and/or ^18^F-fluorodeoxyglucose positron emission tomography (^18^F-FDG PET). At the same time, less patients presented with ventricular tachycardia or fibrillation (VF/VT), impaired left ventricular ejection fraction (LVEF), and elevated brain natriuretic peptide (BNP, >100 ng/L) or N-terminal brain natriuretic propeptide (proBNP, >400 ng/L). The 5-year incidence of death, left ventricular assist device implantation, or VT/VF nearly halved from the group of 1988 to 2009 to the group diagnosed in 2015 to 2019.

### Incidence of CS

The increase found here in the rate of CS diagnoses agrees with comparable data from Sweden^15^ and with similar trends in CS-related hospital admissions^13^ and heart transplantations^14^ in the United States. We emphasize, however, that our data represent the detection rate of clinically manifest CS and cannot be equated with the true incidence of CS as they are tied to the performance of contemporary diagnostics and do not include subclinical cases. Yet, the increase in CS-related heart transplantations cannot be explained by improved clinical diagnostics as the numbers are based on explant studies and a marked proportion of cases (43%) had been missed on pretransplant examinations. Earlier, we reported an increase in the number of postmortem diagnoses of CS between 1998 and 2016 in Finland despite a parallel decrease in the autopsy rate.^5^ The temporally overlapping increases in clinical, explant, and autopsy diagnoses suggest but do not substantiate a genuine change in the incidence of CS. Regarding the epidemiology of sarcoidosis in general, no current reports exist from Finland. Recently, a large study from the Veterans Health Care in the United States showed a significant increase in the incidence and prevalence of sarcoidosis from 2003 to 2019,^19^ while an earlier register study from Sweden revealed stable epidemiologic indexes from 2003 to 2012.^20^

### Diagnostics and the phenotype of CS

The eye-opener in CS diagnostics was the exposure in our country and elsewhere of CS in one-fourth to one-third of middle-aged patients presenting with seemingly idiopathic complete heart block or ventricular tachyarrhythmia.^21^^,^^22^ These and other observations heightened the clinical awareness of CS, promoted the use of advanced cardiac imaging for myocardial inflammation and scarring, and also triggered the launch of the present MIDFIN registry in our country.^1^^,^^3^, ^4^, ^5^ As a consequence, CS likely became recognized earlier in its course with less-severe presenting manifestations and less-extensive myocardial involvement and dysfunction by both imaging and laboratory biomarkers (Tables 1 and 2). Although the present CMRI and ^18^F-FDG-PET data (Table 2) comprehend only one-half or less of our cohort, they show that involvement of either ventricle was less prominent in patients diagnosed in the second half of the 2010s than earlier. Worthy of note, the diagnostic delay was related to the type of cardiac manifestation rather than to the year of diagnosis, with potentially fatal VF and VT apparently triggering initiation of diagnostic studies more promptly than less alarming cardiac symptoms.

The frequency in our cohort of CS proven by myocardial histology, 44%, is higher than the comparable figures, ranging from 6%^23^ to 15%,^10^ in contemporaneous cohorts from the United States^10^^,^^23^ and Japan.^24^ The difference eventuates from our practice of systematically preferring EMB over extracardiac proof of histology in pursuit of definite diagnosis.^16^ The rates of EMB and definite CS declined over time, however, which we assign to the increased use of ^18^F-FDG-PET scans helping identify targets, mediastinal lymph nodes in particular,^25^ for diagnostic extracardiac biopsy. Another likely contributor was the decrease with time in the extent of LV myocardial involvement reducing the sensitivity of EMB.^26^

### Treatment and outcome

Although our data (Table 3) do not expose the dynamics of therapy, they suggest absence of major temporal changes in the care of CS over our study period. The differences observed in the use of immunosuppressive and anti-HF agents likely reflect the more severe phenotype prior to 2010. The rate of ICD implantations remained unaltered over the decades analyzed, but the stability may hide a reduction in the proportion of secondary to primary preventive indications given the drop in VT/VF and the rise in heart block as the presenting manifestation (Table 1). The prognosis of CS improved from the years prior to 2010 to the early and late 2010s, this trend disappearing, however, when differences in the presenting phenotype and the severity of cardiac involvement were considered in statistical analysis. Although it is tempting to attribute the improvement of outcome to earlier start of treatment and surveillance, the contribution of lead time bias, which means that earlier disease detection leads to overestimation of event-free survival, is more than likely. Previous studies have shown that patients with de novo CS have a more severe presenting phenotype and poorer outcome than patients with CS diagnosed on top of known extracardiac sarcoidosis.^23^^,^^27^ In our cohort, the history of extracardiac sarcoidosis at presentation did not vary across the years of diagnosis and was infrequent overall (Table 1).

The incidence of SCD, fatal and aborted combined, was 2.1/100 patient-years in our entire cohort with a decreasing trend over time. Regarding fatal SCDs, the small count found here (n = 11/511) (Table 4) does not represent the entire toll from CS, as our previous study of clinical and cause-of-death registries showed that, for each 1 case of SCD from clinically diagnosed CS, 8 occur concurrently from CS escaping diagnosis until forensic autopsy.^5^ The prevention of SCD is feasible, as shown by the aborted-to-fatal case ratio of 48/11 in our cohort, but the death toll from hidden or clinically missed CS is hard to touch except by pursuing earliest possible diagnosis.

### Strengths and limitations

The strength of our work is the study sample, which is nationwide and large for a rare disease, covers 3 decades of incident cases, adheres to a single set of diagnostic criteria requiring proof of histology,^6^ and includes an exceptionally high proportion of definite CS. The limitations of our cohort are that a recall bias in its creation is possible, and that it does not include subclinical CS and only represents a White population of Northern European ancestry. There was no strictly unified protocol for diagnostics, treatment, and surveillance across the participating hospitals, but all candidates for heart transplantation and selected problem cases were evaluated at the core center (Helsinki University Hospital). The subgroups formed by the years of diagnosis were based on convenience criteria and, due to the study design, had markedly different treatment and follow-up periods that need to be considered in interpreting our prognostic data. The rarity of diagnostic ^18^F-FDG-PET and CMRI studies prior to 2010 is both a key observation and a limitation undermining the comparisons across the subgroups regarding the extent of myocardial involvement. Circulating cardiac troponins and natriuretic peptides had to be analyzed as dichotomized variables because the measurements made by different methods over time could not be transformed into single continuous scales. No independent, external monitoring of source documents was done.

## Conclusions

The detection rate of clinically manifest CS has increased exponentially over the 3 past decades in Finland. At the same time, the phenotype of CS at diagnosis has become milder with less extensive myocardial involvement and dysfunction according to several imaging and laboratory biomarkers. Very likely, clinicians are diagnosing CS at a milder and earlier stage of the disease, this having resulted in an apparent improvement of outcome without major evolution in therapy. Whether the true incidence of CS has changed, instead of the mere detection rate, remains unknown pending further research. Yet, adding the increases in clinical and post-transplant diagnoses found here to the parallel increase in postmortem diagnoses reported earlier^5^ suggests this might be the case.

PERSPECTIVES **COMPETENCY IN MEDICAL KNOWLEDGE:** Familiarity with the varied clinical manifestations of CS and setting a low threshold to the diagnostic use of CMRI and ^18^F-FDG-PET can result in earlier detection and improved prognosis of CS.

**TRANSLATIONAL OUTLOOK:** Sarcoidosis is thought to result from immunologic interactions between environmental triggers and the host’s genetic architecture. Our data raise the possibility that the true incidence of CS has increased over the past few decades. If this can be substantiated, eyes for its cause should be turned to the environment as major alterations in the genetic background are more unlikely.

## Funding support and author disclosures

Dr Pöyhönen was supported by the 10.13039/501100003125Finnish Cultural Foundation (Helsinki, Finland), 10.13039/501100005633Finnish Foundation for Cardiovascular Research (Helsinki, Finland) and Finnish government grant for medical research (Helsinki, Finland). Dr Uusitalo has scientific collaboration with lecture fees with GE Healthcare; and has received lecture fee and advisory board activity with Pfizer. All other authors have reported that they have no relationships relevant to the contents of this paper to disclose.
