# Supplementary material for: 30-Year Trends in the Incidence, Characteristics, and Outcome of Cardiac Sarcoidosis in a Nationwide Cohort
Source: JACC Adv. 2024 Jul 13;3(8):101102. doi: 10.1016/j.jacadv.2024.101102 (PMC11298841; doi:10.1016/j.jacadv.2024.101102)
Supplement: Supplementary data [file mmc1.docx]

**SUPPLEMENTAL APPENDIX**

**SUPPLEMENTAL METHODS**

**Techniques and analyses of cardiac imaging studies**

**Cardiac Magnetic Resonance Imaging (CMRI)**

During the whole study period, pre-diagnostic CMRI studies were performed in 327 of 511 patients (64%) of which available for review were 285 scans (87%). All examinations were conducted on 1.5T or 3.T CMR scanners in the different participating hospitals using phased-array receiver coils and contemporaneous standard protocols^1^ for breath-hold cine studies and late gadolinium enhancement (LGE) imaging. Cine studies were done in long-axis and short-axis planes covering both ventricles, while LGE imaging was done 10 to 15 minutes after an intravenous injection of contrast agent (0.15 mmol/kg) using an inversion‐recovery gradient-echo sequence in views identical to cine imaging. The CMRI studies were evaluated at the core center (Helsinki University Hospital) by a single CMR-trained cardiologist (P.P.) blinded to clinical data. Left and right ventricular (LV and RV) volumes and LV mass were assessed using standard protocols^2^ with papillary muscles and outflow tract included in the LV volume. The presence of LGE was assessed visually. The extent of LGE as a percentage of LV mass was assessed using the full width at half maximum method.^3^ Image analyses were performed using QMass MR software® (version 8.1, Medis Medical Imaging Systems, Leiden, the Netherlands).

**^18^F-fluorodeoxyglucose Positron Emission Tomography (^18^F-FDG-PET)**

^18^F-FDG-PET scans were performed and available in 231 patients (45%). The PET images were quality-checked and read retrospectively by a core lab nuclear medicine physician (V.U). The 191 studies (83%) with acceptable quality and without confounding physiologic FDG uptake due to diet failure were included for further semi-quantitative analysis. All studies had been preceded by dietary manipulation to switch off myocardial glucose metabolism, but the preparations varied both between institutions and over the years covered by our research. Fasting for at least 12 hours prior to ^18^F-FDG administration was the minimum requirement, and routine use of a high-fat, low-carbohydrate diet was adopted at Helsinki University Hospital in 2013. Imaging was done 60 minutes after ^18^F-FDG injection with the standard clinical PET/CT systems available locally in the participating hospitals.

PET analyses were done retrospectively for research use only using Syngo.Via (Siemens Healthcare GmbH, Erlangen, Germany) and Hermes (Hermes Medical Solutions, Stockholm, Sweden) softwares. The number of LV segments with abnormal focal or focal-on-diffuse ^18^F-FDG uptake was summed according to the AHA 17-segment model.^4^

**Single-photon Emission Tomography Imaging (SPECT)**

SPECT perfusion scans at rest were performed and available in 141 patients (28%), with acceptable quality in 136 cases (96%). All perfusion studies were obtained using standard dual-headed gamma cameras, and ^99m^Technetium-labeled sestamibi or tetrofosmin were used as the radiotracers. The extent of the myocardial scars was retrospectively quantified by a nuclear medicine physician (V.U) at the core imaging center as a summed rest score (SRS) according to the AHA 17 segment model as previously described. ^4,5^ Hermes software (Hermes Medical Solutions, Stockholm, Sweden) was used for the image analysis.

**REFERENCES**

1. Kramer CM, Barkhausen J, Flamm SD, Kim RJ, Nagel E; Society for Cardiovascular Magnetic Resonance Board of Trustees Task Force on Standardized Protocols: Standardized cardiovascular magnetic resonance imaging (CMR) protocols Society for Cardiovascular Magnetic Resonance (SCMR) board of trustees task force on standardized protocols. J Cardiovasc Magn Reson. 2008;10:35.
2. Schulz-Menger J, Bluemke DA, Bremerich J, et al. Standardized image interpretation and post-processing in cardiovascular magnetic resonance: Society for Cardiovascular Magnetic Resonance (SCMR) board of trustees task force on standardized post-processing. J Cardiovasc Magn Reson. 2013;15:35.
3. Flett AS, Hasleton J, Cook C, et al. Evaluation of techniques for the quantification of myocardial scar of differing etiology using cardiac magnetic resonance. JACC Cardiovasc Img. 2011; 4:150-156.
4. Cerqueira MD, Weissman NJ, Dilsizian V, et al. American Heart Association Writing Group on Myocardial Segmentation and Registration for Cardiac Imaging. Standardized myocardial segmentation and nomenclature for tomographic imaging of the heart. A statement for healthcare professionals from the Cardiac Imaging Committee of the Council on Clinical Cardiology of the American Heart Association. Circulation. 2002;105:539-42.
5. Hesse, B, Tägil, K, Cuocolo, A, et al. EANM/ESC procedural guidelines for myocardial perfusion imaging in nuclear cardiology. Eur J Nucl Med Mol Imaging. 2005; 32: 855–897.
